# 

**Published:** 1920-07-31

**Authors:** 


Messrs. Siemens Brothers & Co., Ltd., are taking over the lamp and supplies department of Messrs. Siemens Brothers Dynamo Works, Ltd. Contemporaneously with this change the company is making provision for a largely increased output in metal filament lamps, and it is announced that henceforth these lamps will be sold by the company under the distinctive trade name of "Xcel." This trade name will apply to all types of metal filament lamps manufactured by the company.

Mr. George Paxton, the well-known manufacturing optician, has left £250 to the Royal Eye Hospital, £200 each to the Central London Ophthalmic Hospital, the Western Ophthalmic Hospital, and the Bristol Eye Hospital, £150 to the Bristol Eye Dispensary, and £100 each to King's College, St. Thomas's, Middlesex, University College, and the Elizabeth Garrett Anderson Hospitals, providing that the business relations with his firm, Messrs. Curry & Paxton, subsisting at the date of his will exist.

### Passing Events and Topics.

### A Children's Hospital for Bombay.

There is a movement on foot to establish a children's hospital as a permanent memorial of the approaching visit of the Prince of Wales to India. The proposal is a good one, and it is only fitting that Bombay, which has taken the lead in the infant-welfare agitation which has now spread throughout India, should be the first great Indian city to establish a special hospital for children.

### The Red Cross in India.

A Red Cross Society for India has now been established by decree of the Legislative Council. The Bill was introduced during the current session in view of the urgent importance of placing the Indian Society in a position to deal with the numerous references which are already being received from the League of Red Cross Societies, and also with the object at the earliest possible juncture of framing the regulations enabling provincial branches to place their organisations upon a satisfactory basis.

### Personalia.

Sir William Osler, Bart, F.R.S., late Regius Professor of Medicine in the University of Oxford, left estate of the gross value of £15,865. He left his medical and scientific library to the Medical Faculty of the McGill University, Montreal, and all his other property to his wife. At her death, or earlier if she should wish, his residence, 13 Norham Gardens, Oxford, is to be given to the Dean, Canons, and Governing Body of Christ Church as the residence of the Regius Professor of Medicine.

Dr. John Wall, M.B., B.Ch., of Manchester, has been appointed second assistant medical officer at Cardiff Mental Hospital.

Mr. Harold B. Scargill, M.B., B.S.(Lond.), has been appointed medical officer in charge of the radiological department, and Mr. Fredk. J. Stansfield, M.R.C.S., L.R.C.P., medical officer in charge of the electro- and radio-therapeutic department of the General Infirmary, Leeds.

### Matrons' and Sisters' Appointments.

Infirmary, Holgate, Linthorpe, Middlesbrough.—The following have been appointed sisters: Misses Ward, Bilton, Harrison, and Barnes. The Misses Ward and Bilton were trained at Wakefield Poor-Law Infirmary, where they later held the position of sister. Miss Harrison was trained at Newcastle Poor-Law Infirmary, and Miss Barnes at the Infirmary, Epsom. They all hold the certificate of the Central Midwives Board.

Queen Charlotte's Lying-in Hospital, Marylebone Road.—Miss Florence Rodwell has been appointed district sister-superintendent. She was trained at Lincoln County Hospital and with the Maternity Nursing Association, and she has been matron of a maternity home at Hull.

Royal Lancaster Infirmary.—Miss K. G. Lloyd, R.R.C., has been appointed matron. She was trained at the General Hospital, Birmingham, where she has since been ward sister and assistant matron, and has been matron of the 1st Southern General Hospital, Edgbaston, Birmingham.

Allt-yr-yn Hospital, Newport, Mon.—Miss E. M. Adams has been appointed night sister. She was trained at the General Hospital, Birmingham, and at the City Fever Hospital, Birmingham, and has been sister at the City Hospital, Lodge Moor, Sheffield, Shoreditch Infirmary, and of the Kent and Canterbury Hospital. Miss Adams holds the certificate of the Central Midwives Board.

### The College of Nursing (Limited by Guarantee).

### EDINBURGH CENTRE.

By the kind invitation of Miss Thyne, a garden-party for the members of the Edinburgh Centre of the College of Nursing was held in the grounds of West House, Morningside, on the afternoon of Wednesday, July 21. The weather was all that could be desired, and a most enjoyable afternoon was spent.

### SCOTTISH BOARD.

The office of the Scottish Board at 8 Drumsheugh Gardens, Edinburgh, will be closed during the month of August.

### BIRMINGHAM THREE COUNTIES CENTRE.

On Thursday, July 22 (by kind permission of Mrs. Shirley-Smith), a most successful American Tea was held at 206 Hagley Road in aid of the printing expenses in connection with the approaching Scenic Fair. A large attendance of nurses and their friends was a practical demonstration of their appreciation of the efforts being made to establish a Club and Lecture Room in their midst.

On Tuesday, July 27, Dr. G. B. Dixon, Tuberculosis Officer to the city, gave an interesting lecture on "Tuberculosis." In the course of his address Dr. Dixon referred to the causes of pre-disposition, the channels of infection, treatment by heliotherapy, induction of artificial pneumothorax and after-effects. At the conclusion of the lecture he kindly arranged for any nurses present to be shown over Yardley Road Sanatorium on Saturday next at 3 p.m.

King Edward VII. Hospital at Cardiff mourns the loss of two of its most respected medical officers—Dr. Rhys Griffiths and Dr. Russell Thomas. The former had been associated with the institution altogether for thirty-eight years, thirty-four of which as a member of the honorary surgical staff. During the war he worked very hard, both at the King Edward and the 3rd Western General Hospitals, and undoubtedly overtaxed his strength. Tributes to the qualities of the doctors were paid at a meeting of the Board of Management of the King Edward Hospital, when a vote of condolence with the relatives was passed, all present standing.

### ENQUIRIES AND ANSWERS.

### *EMPLOYMENT AND TRAINING.*

### Nursing in New Zealand.

We regret we are unable to give you the addresses you require, but if you will write to the High Commissioner for New Zealand, 415 Strand, London, W.C. 2, he will probably be able to help you in this matter. A coupon should be enclosed with each inquiry; we cannot give postal replies.—R. G. C.

### Ambulance Nurse or Seamstress.

We suggest that you write to the St. John Ambulance Association, St. John's Gate, Clerkenwell, E.C., and the Metropolitan Asylums Board, Embankment, W.C., with regard to obtaining a post as ambulance nurse. Watch the advertisements which appear from time to time in the nursing journals and daily press for a post as seamstress in an institution. We cannot give postal replies; a coupon must be sent with each query.—A. C.

### Nursing Abroad.

We strongly advise you to obtain a post before going abroad, unless you are in a position to go out on the chance of something turning up. We regret we do not know of any vacancies in works or institutions abroad other than those that may be advertised in the Press. Why not write to Messrs. Ford, Ltd., the firm that you mention, and inquire if they have any vacancies? We give you a few addresses to which you might write in regard to obtaining a post in hospital: The Secretary, South African Colonisation Society, 2 Army and Navy Mansions, Victoria Street, London, S.W. 1; The Matron, General Public Hospital, St. John, New Brunswick; the Matron, Victoria Public Hospital, Fredericton, New Brunswick; the High Commissioner for New Zealand, 415 Strand, W.C. 2. If you would care for Bush nursing, write to the Secretary of the Bush Nursing Association, Twyford House, 17 Castlereagh Street, Sydney, N.S.W. We do not give postal replies; a coupon should be enclosed with each inquiry.—A. L. S.

### Recognised Training Schools.

We give you the names of some recognised training-schools in the localities you mention: The Royal Infirmary, Sunderland; Sunderland Union Infirmary, Hylton Road, Sunderland; North Staffordshire Infirmary and Eye Hospital, Hartshill, Stoke-upon-Trent; Harton Hospital, South Shields; Royal Salop Infirmary, Shrewsbury; and the Mansfield and District Hospital. Your only course is to write to the Matron of the respective hospitals and ask if there is a vacancy for a probationer.—Pat.

### Club for Nursing Sisters.

The proposed new club for nursing sisters of the Services is not yet complete, but is materialising rapidly. The temporary offices are at 35 Mecklenburgh Square, and we suggest that you write to the Secretary and make your inquiries. The scheme embraces members of the Q.A.I.M.N.S., Q.A.R.N.N.S., the Reserves, T.F.N.S., the Indian Nursing Service, and probably nursing sisters of the Ministry of Pensions Service and the B.R.C.S.—Nursing Sister.

### Nursing in Palestine.

The Edinburgh Medical Missionary Society has hospitals in Nazareth and Damascus, and we suggest that you write to the Secretary at 56 George Square, Edinburgh, who will possibly be able to help you.—F. V.

### *MISCELLANEOUS.*

### Book on Gynæcology.

You will, no doubt, find "Gynæcology for Nurses and Gynæcological Nursing" of great help to you. It is by Comyns Berkeley, M.A., M.D., and is published by The Scientific Press, Ltd., 28 and 29 Southampton Street, Strand, W.C. 2. The price is 6s. 4d. post free.—Nurse.

### Fish Supply.

Messrs. Bilbrough, Ltd., of 51 Hamilton Dock, Lowestoft, are offering London hospitals daily deliveries of fish from the dock on favourable terms. Several hospitals are testing the offer, and provided that the recently improved transport arrangements secure punctual delivery, the scheme should be successful. Supplies of fish at reasonable prices are sometimes so difficult to secure that the offer is worth a trial.—W. H.

